# Herbivore associated elicitor-induced defences are highly specific among closely related *Nicotiana* species

**DOI:** 10.1186/s12870-014-0406-0

**Published:** 2015-01-16

**Authors:** Shuqing Xu, Wenwu Zhou, Sarah Pottinger, Ian T Baldwin

**Affiliations:** Department of Molecular Ecology, Max Planck Institute for Chemical Ecology, Hans-Knöll-Straße 8, D-07745 Jena, Germany

**Keywords:** Specificity of herbivore induced defence, *Nicotiana*, Jasmonic acid, Trypsin proteinase inhibitor, Induced resistance, Specialist and generalist

## Abstract

**Background:**

Herbivore-induced defence responses are often specific - different herbivores induce different defence responses in plants - and their specificity is largely mediated by chemical cues (herbivore-associated elicitors: HAEs) in insect oral or oviposition secretions. However, the specificity and the mechanisms of HAE-induced defence have not been investigated in the context of the evolutionary relationships among plant species. Here we compare the responses of six closely related *Nicotiana* species to a synthetic elicitor, *N*-linolenoyl-glutamic acid (C18:3-Glu) and HAE of two insect herbivores (the Solanaceae specialist *Manduca sexta* and generalist *Spodoptera littoralis*).

**Results:**

HAE-induced defences are highly specific among closely related *Nicotiana* species at three perspectives. 1) A single *Nicotiana* species can elicit distinct responses to different HAEs. *N. pauciflora* elicited increased levels of JA and trypsin proteinase inhibitors (TPI) in response to C18:3-Glu and the oral secretions of *M. sexta* (OS_*Ms*_) but not to oral secretions of *S. littoralis* (OS_*Sl*_). In contrast, *N. miersii* only responded to OS_*Sl*_ but not to the other two HAEs. The specific responses to different HAEs in *Nicotiana* species are likely due to the perception by the plant of each specific component of the HAE. 2) One HAE can induce different defence responses among closely related *Nicotiana* species. OS_*Ms*_ and C18:3-Glu induced JA and TPI accumulations in *N. linearis*, *N. attenuata*, *N. acuminata* and *N. pauciflora*, but not in *N. miersii* and *N. obtusifolia*. 3) The effect of HAE-induced defences differ for the Solanaceae specialist *M. sexta* and the generalist *S. littoralis*. Among the four tested *Nicotiana* species, while the growth rate of *M. sexta* was only reduced by the induced defences elicited by C18:3-Glu; the growth rate of *S. littoralis* can be reduced by the induced defences elicited by all three HAEs. This is likely due to differences in the susceptibility of the specialist *M. sexta* and generalist *S. littoralis* to induced defences of their host.

**Conclusions:**

Closely related *Nicotiana* species elicit highly specific defence responses to herbivore associated elicitors and provide an ideal framework for investigating the molecular mechanisms and evolutionary divergence of induced resistance in plants.

**Electronic supplementary material:**

The online version of this article (doi:10.1186/s12870-014-0406-0) contains supplementary material, which is available to authorized users.

## Background

Induced defences are widespread in plants and play an important role for plant fitness [1]. In response to herbivore attack, plants distinguish mechanical damage from damage caused by feeding insects through the perception of chemical cues (herbivore-associated elicitors: HAEs) in insect oral secretions (OS) [2]. Such HAE-induced plant defences have frequently been shown to be insect species-specific [3-6]. This is because elicitors from different herbivore species vary both qualitatively and quantitatively and the ability to respond to HAEs varies among plant species [4,6].

The specificity of HAE-induced plant responses appear at multiple levels. The phytohormone jasmonic acid (JA) and its derivatives play a central role in the activation of defences against most insect herbivores [7,8]. The induced accumulation of JA in plants can be HAE-specific. For example, in eggplant two fatty acid amino acid conjugates (FACs), volicitin and *N*-linolenoyl-Gln, induced more than a two-fold increase in JA levels in comparison with wounding alone. However two other HAEs tested, caeliferin A16:0 and inceptin, did not induce JA accumulation [6]. This suggests that the specificity of HAE-induced JA accumulation is probably mediated by specific receptor-ligand interactions, although the molecular mechanisms remain to be understood. The specificity of induced JA accumulation among different HAEs can also be mediated by hormonal crosstalk [4,9,10]. In *N. attenuata*, *Spodoptera exigua* oral secretion (OS_*Se*_) induced lower levels of JA accumulation than *Manduca sexta* oral secretion (OS_*Ms*_). This is due to enhanced glucose oxidase (GOX) activity in *S. exigua* OS eliciting a salicylic acid (SA) burst which attenuates JA induction [11]. Furthermore, different plant species can display JA responses with a different timing and/or magnitude after exposure to the same HAE. While volicitin induces JA accumulations in maize, eggplant and soybean, this elicitor does not induce JA accumulations in *Arabidopsis thaliana* and cowpea [6]. This indicates that the HAE-induced JA response varies among different plant families. However, the variation of such a response among closely related species and the extent to which its specificity is mediated by receptor-ligand interaction [12] or hormonal cross-talks is largely unknown.

In addition to promoting phytohormone accumulation, HAE stimulation can result in accumulation and mobilisation of defence compounds, such as trypsin proteinase inhibitors (TPI) and diterpene glycosides (DTG) which function as direct defences [13,14]. Because most HAE-induced metabolomic responses are thought to be mediated by JA [13,15,16], the specificity of the HAE-induced response is thought to be largely associated with the specificity of HAE-induced JA accumulations. However, other HAE-induced phytohormones can also fine-tune induced metabolomic responses [17].

Certain induced plant defences can reduce the growth and fecundity of insect herbivores, but the effect of an induced defence on insect growth varies among insect species [18,19]. As a generalisation, specialist herbivores tend to have a higher tolerance for the toxins produced by their host plant than do generalist herbivores [20]. Therefore, specialist herbivores are on average less sensitive to the changes of their host plant defences than generalists are [20]. Thus it is thought that the induced resistance to a specialist herbivore is more specific (elicited by only a few specific HAEs) than the induced resistance to a more generalist herbivore (elicited by larger number of different HAEs). However, this hypothesis has not been systematically tested using multiple species and different HAEs [20].

The specificity of induced defence at the phytohormone, metabolite and herbivore performance levels have been investigated in different plant systems [5,6,18]. However, these responses were usually studied separately. It is challenging to integrate the specificity of induced defences at different levels and to understand the underlying mechanisms as they vary amongst different plants [6]. In this study we used a comparative approach to investigate the specificity of the induced responses to three different HAEs among six closely related *Nicotiana* species at multiple levels, including phytohormones, metabolites and herbivore performance. We specifically focused on three questions: 1) to what extent does one plant species respond to different HAEs? 2) to what extent does one HAE induce different responses among closely related plant species; and 3) does one HAE-induced defence affect insect herbivores differently?

**Figure 1:**
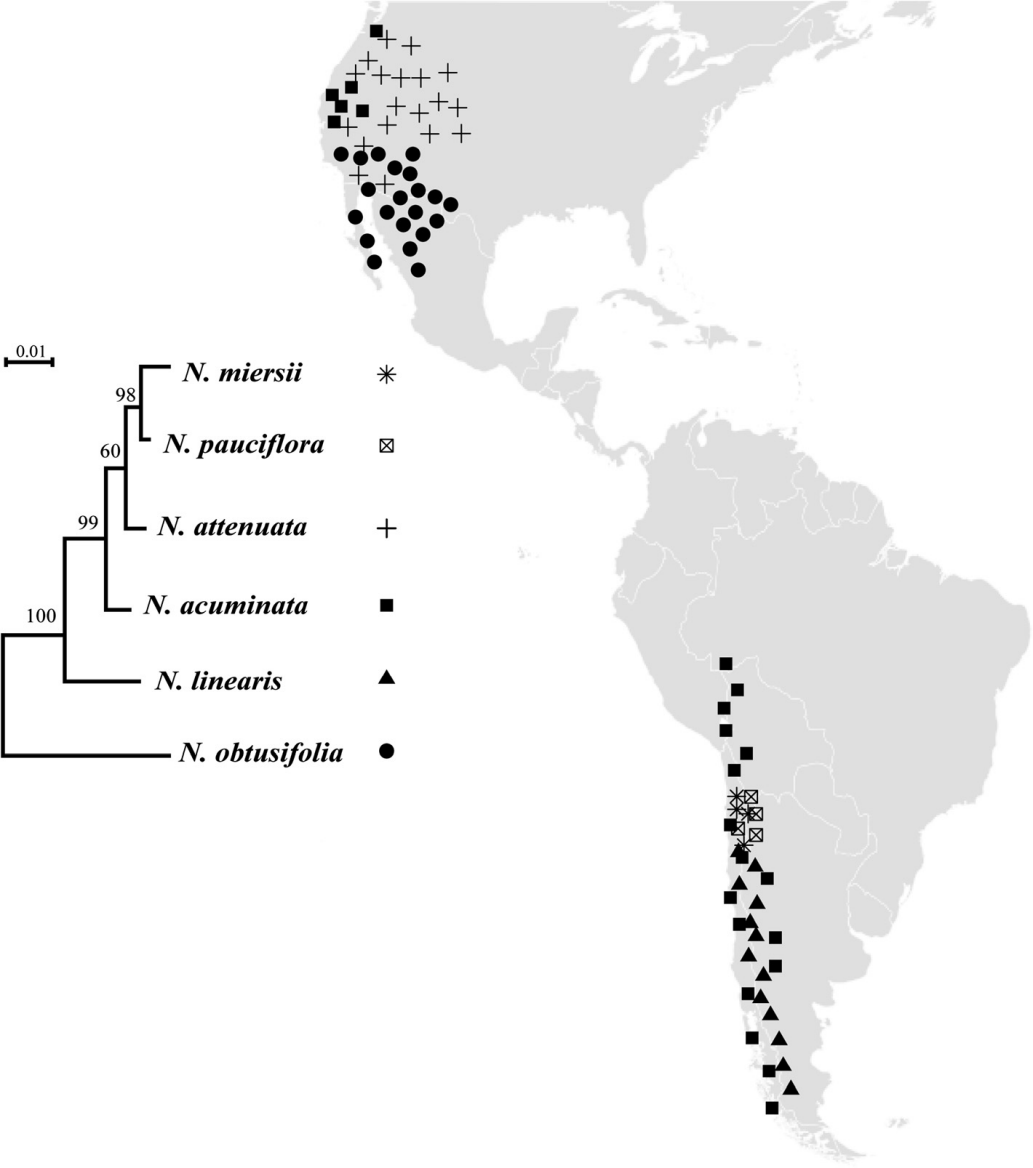
**The geographic distribution of six closely related** ***Nicotiana*** **species analysed in this study, redrawn from Goodspeed T. H. [** 23 **].** A phylogenetic tree of the six *Nicotiana* species was constructed from partial nepGS gene sequences obtained from Clarkson et al. 2010 [24], using maximum likelihood method and numbers on each branch represent bootstrap value. Each symbol represents different species. The location of the symbol indicates the distribution of the species that was extracted from Goodspeed T. H [23]. Filled circle: *N. obtusifolia*; filled triangle: *N. linearis*; filled square: *N. acuminata*; plus: *N. attenuata*; square with cross inside: *N. pauciflora*; star: *N. miersii.*

The three HAEs used in this study are: the oral secretions of *M. sexta* (OS_*Ms*_, a specialised feeder on solanaceous plants), the oral secretions of *Spodoptera littoralis* (OS_*Sl*_, a generalist plant feeder) and *N*-linolenoyl-glutamic acid (C18:3-Glu, a fatty acid conjugate (FAC) which has been shown to be the most active elicitor in *Manduca sexta* oral secretions) [21]. The concentration of FACs, including C18:3-Glu, in OS_*Sl*_ is 500 times lower than that of OS_*Ms*_ [22]. With these three HAEs we investigated the specificity of induced defence responses in six closely related, diploid (2n = 12) *Nicotiana* species that are widely distributed across North and South America: *Nicotiana obtusifolia*, *N. linearis*, *N. acuminata*, *N. attenuata*, *N. pauciflora* and *N. miersii* [23] (Figure 1). All species are annuals from the *Petunioides* clade, except *Nicotiana obtusifolia* which is a perennial plant from the sister clade *Trigonophyllae* [24]. Within these six closely related species, *N. attenuata* is an ecological model plant in which the molecular and ecological mechanisms of sophisticated herbivore-induced defence have been studied in detail. Here we use a comparative approach to study the specificity of induced defence from both mechanistic and evolutionary perspectives.

## Results

### Induction of JA is both HAE- and species-specific in *Nicotiana*

**Figure 2:**
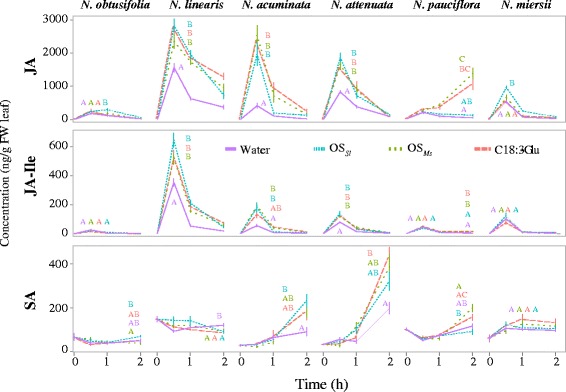
**HAE induced phytohormone responses in the different** ***Nicotiana*** **species is both HAE- and species-specific.** Each column indicates a different species. The top row represents induced JA responses, the middle row represents induced JA-isoleucine (JA-Ile) responses and the bottom row represents induced salicylic acid (SA) responses. Phytohormones were measured at 0 h, 0.5 h, 1 h and 2 h after HAE induction. Different line types represent different treatments. Solid purple colour indicates wounding (W) + water, light blue dotted line indicates W + *S. littoralis* OS (OS_*Sl*_) induction; green dashed line indicates W + *M. sexta* OS (OS_*Ms*_) induction, and vermillion dashed line indicates W + C18:3-Glu induction. Letters indicate the statistically significant differences (*p* < 0.05) at the peak JA, JA-Ile and SA concentrations for each species as determined by ANOVA or Kruskal-Wallis test depending on whether the data were normally distributed or not. The error bar indicates standard error.

The pattern of JA accumulation was specific for each HAE studied and the HAE-induced JA accumulation pattern also differed among the *Nicotiana* species (Figure 2). The analysis revealed that JA accumulation in response to wounding and HAE addition was highly plant species- and HAE-specific. The highest plant species-specificity of HAE-induced JA induction was found in *N. obtusifolia* and *N. pauciflora* (Figure 2). In *N. obtusifolia*, neither C18:3-Glu nor OS_*Ms*_ affected JA levels, and only OS_*Sl*_ induced a slight accumulation of JA which was not reflected in higher JA- isoleucine (JA-Ile) levels at 1 h after elicitation. In contrast to the other five *Nicotiana* species that showed the highest JA levels at 30 minutes, *N. pauciflora* showed peak JA levels at 2 h after HAE induction (Figure 2).

**Figure 3:**
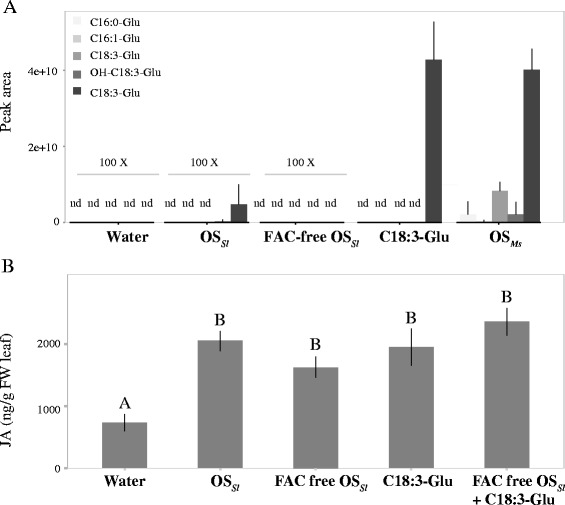
***S. littoralis*** **oral secretion (OS) induces JA accumulation independent of the FAC content of the OS. A**: The five most abundant FAC compounds were measured by HPLC-MS for all HAEs and control used in this study. Water; oral secretion from *S. littoralis* (OS_*Sl*_); OS_*Sl*_ with fatty acid amino acid conjugates (FAC) removed (FAC-free OS_*Sl*_) using an ion exchange column; C18:3-Glu and oral secretion from *M. sexta* (OS_*Ms*_). Each shading represents a specific FAC compound. Y-axis shows the peak area of the target molecular ion of each compound. For each HAE, six replicates were used. Error bar indicates the standard deviation. “nd” refers to samples in which an FAC was not detected. **(B)** The induced JA accumulation in *N. attenuata* induced by wounding + oral secretion of *S. littoralis*. Y-axis shows the JA concentration at 30 minutes after induction and axis refers to different HAEs that were added to leaf punctures. Letters indicates the significance of differences (*p* < 0.05, *post hoc* Tukeyhonest significant test after ANOVA).

The specificity HAE was evident when comparing JA responses to the elicitations with OS_*Ms*_ and OS_*Sl*_. Elicitations with OS_*Ms*_ and C18:3-Glu showed similar JA and JA-Ile induction patterns among all six species, which is consistent with a previous study that found C18:3-Glu to be the main component in OS_*Ms*_ that elicits JA accumulation in *Nicotiana*. The strongest differences between OS_*Ms*_ and OS_*Sl*_ induced JA accumulations were found in *N. pauciflora* and *N. miersii* (Figure 2). In *N. miersii*, neither OS_*Ms*_ nor C18:3-Glu induced any JA accumulations (Figure 2), but OS_*Sl*_ was highly effective, eliciting JA increases 1.8 times that of a wounding control (wounding + water). This indicates that while C18:3-Glu elicitation failed to induce JA, *N. miersii* specifically responds to OS_*Sl*_. Interestingly, the opposite pattern was found in *N. pauciflora*, in which OS_*Sl*_ did not induce JA and JA-Ile increases, while both OS_*Ms*_ and C18:3-Glu induced a more than 20-fold JA increase in comparison to controls (wounding + water). These results suggest that OS_*Sl*_ may contain FAC-independent elicitors and/or inhibitors. To test this hypothesis, all FACs were removed from OS_*Sl*_ using an ion exchange column and the FAC-free OS_*Sl*_ was applied to wounds in *N. attenuata* leaves, a species showing induced JA accumulation in response to both FAC and OS_*Sl*_ (Figure 2). The results revealed that FAC-free OS_*Sl*_ can elicit significant JA accumulations in *N. attenuata* at 30 minutes after treatment confirming the existence of FAC-independent elicitors in OS_*Sl*_ (Figure 3A and B). Furthermore, adding synthetic FAC to FAC-free OS_*Sl*_ produced a treatment which elicited a similar increase in JA accumulation as that produced by FAC alone, suggesting that OS_*Sl*_ lacks factors which inhibit FAC responses (Figure 3B). The same result was observed for *N. pauciflora*, in which FAC-complemented OS_*Sl*_ induced similar JA accumulations to that elicited by FAC and OS_*Ms*_ (Additional file 1). These results suggest that OS_*Sl*_ and OS_*Ms*_ have different elicitor compositions which induce JA accumulations in *Nicotiana* through different molecular mechanisms. The induced SA levels did not show an HAE-specific pattern (Figure 2).

### The induction of trypsin proteinase inhibitor (TPI) activity differs across different *Nicotiana* species

**Figure 4:**
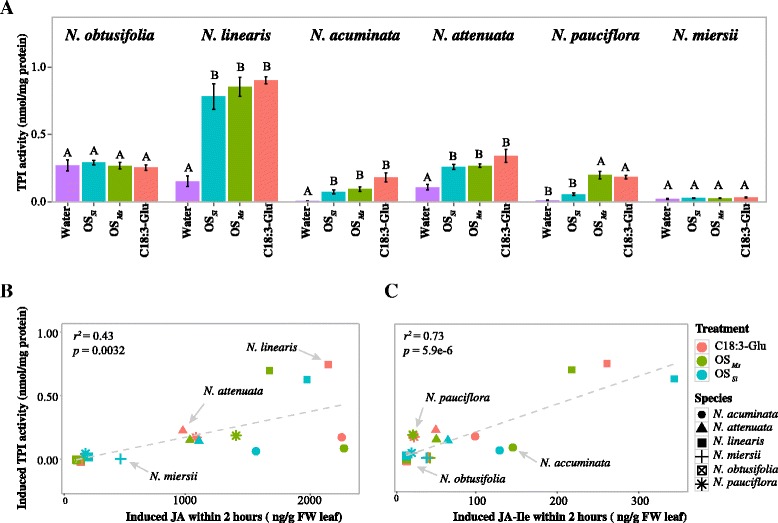
**HAE-induced TPI activity is species-specific and correlated with induced levels of JA and JA-Ile. A**: TPI activity was measured 24 h after induction. Letters indicate statistical significance among different treatments (*p* < 0.05, *post hoc* Tukey honest significant test after ANOVA). **B** and **C**: the correlation between induced TPI activity and induced JA **(B)** and JA-Ile **(C)** accumulation within 2 h. The induced TPI activity, JA and JA-Ile accumulation were calculated as Euclidean distance between control samples (wounding + water) and each HAE treated samples. The linear regression was calculated and shown in the figure. Each colour refers to different treatments: black, wounding + C18:3-Glu; dark grey, wounding + OS_*Ms*_; light grey, wounding + OS_*Sl*_. Each symbol refers to different species: Filled circle, *N. obtusifolia*; filled triangle, *N. linearis*; filled square, *N. acuminata*; plus, *N. attenuata*; square with cross inside, *N. pauciflora*; star, *N. miersii.*

The HAE-induced TPI activity varied among the six species investigated (Figure 4A) and correlated with the induction of JA and JA-Ile accumulation (Figure 4B and C). Two species, *N. miersii* and *N. obtusifolia,* which showed only low levels of JA and JA-Ile accumulations within 2 h after HAE induction, did not show TPI activity changes in comparison to the control at 24 h after treatment. The other four species, *N. linearis*, *N. acuminata*, *N. attenuata*, and *N. pauciflora,* which showed a high level of JA and JA-Ile accumulation within 2 h after HAE induction, also showed high induced TPI activity at 24 h after treatment. All three HAEs induced similar levels of TPI activity and this was consistent among all species with the exception of *N. pauciflora,* where TPI activity was induced by OS_*Ms*_ and C18:3-Glu but not by OS_*Sl*_. This also correlates with the observation that only OS_*Ms*_ and C18:3-Glu induced JA and JA-Ile accumulations in *N. pauciflora* within 2 h after treatment. Overall HAE-induced TPI activity is highly correlated with the level of induced JA and JA-Ile accumulations and therefore shows a response pattern specific to each *Nicotiana* species (Figure 4).

### HAE-induced resistance to *M. sexta* and *S. littoralis* are HAE-specific, but only induced resistance to *M. sexta* is species-specific

**Figure 5:**
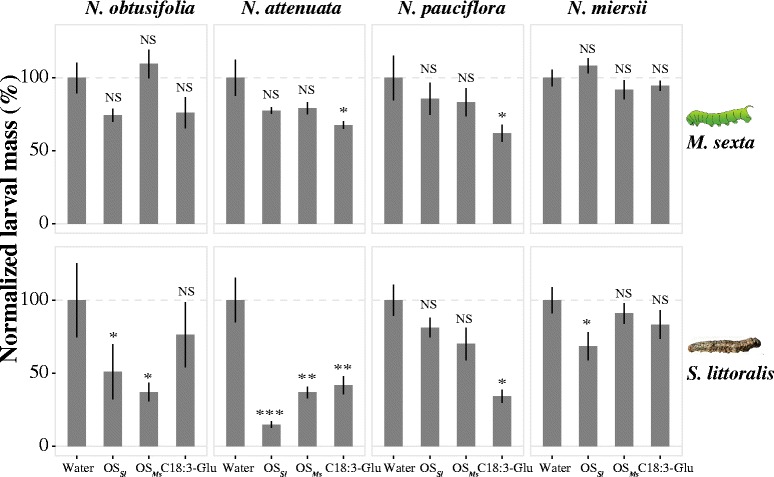
**HAE-induced resistance to** ***M. sexta*** **and** ***S. littoralis*** **in four** ***Nicotiana*** **species.** Each column represents different species, top and bottom rows represent induced resistance to *M. sexta* and *S. littoralis* respectively. For each plant species, the larva mass of *M. sexta* and *S. littoralis* fed on HAE-treated leaves was normalized to *M. sexta* and *S. littoralis* fed on leaves treated with wounding + water (control). Each bar indicates insects fed on leaves treated with wounding and different HAEs. Symbols on each bar represent the statistical significance of each treatment to control. NS indicate no significance was found (*p* > 0.05); * indicates *p* < 0.05; ** indicates *p* < 0.01; *** indicates *p* < 0.001.

A detached leaf assay was performed to measure the induced resistance of four *Nicotiana* species that showed HAE and species-specific phytohormone responses to the specialist herbivore *M. sexta* and the generalist *S. littoralis*. Induced resistance to *M. sexta* was found to have high species- and HAE-specificity. Among the four tested species only two, *N. attenuata* and *N. pauciflora*, showed induced resistance to *M. sexta* suggesting this induced resistance is species-specific. Interestingly, in both species, only *M. sexta* larvae fed on C18:3-Glu treated leaves gained less mass than those fed on leaves treated with water (control) (Figure 5). The larval mass of *M. sexta* fed on OS_*Ms*_ or OS_*Sl*_ treated leaves was not significantly different from the control, indicating the induced resistance is specific to C18:3-Glu.

Different from the induced resistance to *M. sexta*, the induced resistance to *S. littoralis* was found only to be HAE-specific. The induced resistance to *S. littoralis* can be observed in all four species suggesting low species specificity. However, within a species, the level of induced resistance to *S. littoralis* differed amongst the different HAEs (Figure 5), indicating high HAE-specificity within species. In summary, our results showed that the induced resistance to *M. sexta* differs from the induced resistance to *S. littoralis*.

## Discussion

The specificity of HAE-induced defences can be analysed from at least three perspectives: 1) induced defences of one plant species in response to different HAEs; 2) induced defences of different plant species in response to the same HAE; and 3) the effect of the same induced defences on different herbivorous insects. Here we investigated the specificity of HAE-induced defences from all three perspectives.

### To which extent does one plant species respond to different HAEs?

Plants can respond to different HAEs at multiple levels. At the phytohormone level, the six *Nicotiana* species displayed the same JA responses to C18:3-Glu as to OS_*Ms*_ but not to OS_*Sl*_ (Figure 2). This is consistent with a previous study which showed that C18:3-Glu is the elicitor responsible for JA accumulation in OS_*Ms*_ [21]. Differences in JA accumulation between OS_*Sl*_ and the other two HAEs are likely due to the different elicitors in OS_*Sl*_ capable of inducing JA accumulation. *N. obtusifolia* and *N. miersii* showed no JA responses to C18:3-Glu and OS_*Ms*_, which is consistent with previous studies [25,26], but they did accumulate JA in response to OS_*Sl*_. In contrast, *N. pauciflora* showed a 20-fold JA increase in response to C18:3-Glu and OS_*Ms*_ at 2 h after induction but no JA increase in response to OS_*Sl*_. These results indicate that the molecular mechanisms of OS_*Sl*_ perception and subsequent JA responses are likely different from those of OS_*Ms*_/C18:3-Glu perception and responses, since a plant species can lose its perception of one but maintain sensitivity towards others. Indeed our results show that OS_*Sl*_ induced JA accumulation is independent of FAC, because OS_*Sl*_ and the FAC-free OS_*Sl*_ induced the same level of JA accumulation in *N. attenuata* (Figure 3A and B). In addition, OS_*Sl*_ and FAC-supplemented OS_*Sl*_ induced similar levels of JA in both *N. attenuata* and *N. pauciflora.* This suggests that the lack of response to OS_*Sl*_ at the JA level is not due to the presence of an inhibitor in OS_*Sl*_ (Figure 3 and Additional file 1). Studies have shown that plants can respond to several non-FAC elicitors in the OS_*Sl*_, such as porin-like proteins (PLP) [27], and oligosaccharides [28]. However, whether these elicitors trigger JA accumulation in *Nicotiana* species remains unknown. Interestingly, the interaction of JA and SA did not show an HAE-specific pattern (Figure 2). In summary, *Nicotiana spp.* showed highly-specific induced JA accumulation in response to different HAEs due to their different elicitor compositions and we hypothesize that this may be mediated by differences in receptor-ligand interactions amongst the species.

TPI has been shown to be an important anti-herbivore defence trait. Most *Nicotiana* species showed similar levels of induced TPI activity in response to the different HAEs at 24 h after induction (Figure 4A). This suggests that induced TPI activity is not HAE-specific, although further studies on the complete kinetics of HAE-induced TPI activity in all six *Nicotiana* species are required to confirm this inference. The only exception was found in *N. pauciflora*, which showed increased TPI activity in response to elicitation by C18:3-Glu and OS_*Ms*_ but not OS_*Sl*_ (Figure 4A). We conclude that this is largely due to the low level of OS_*Sl*_-induced JA and JA-Ile accumulation in this species (Figure 2, 5th column), because studies have shown that the induction of TPI activity is regulated by JA-signalling [15,29]. A significant within-species correlation between specific HAE induction of JA and the corresponding TPI activity was not found in *N. miersii* and *N. obtusifolia*. These species showed induced JA accumulations only in response to OS_*Sl*_ (Figure 2, 1st and 6th column) and no induced TPI activity at all (Figure 4A). This is likely because of the low levels of OS_*Sl*_-induced JA and JA-Ile in *N. obtusifolia* and *N. miersii* and such low levels of JA/JA-Ile may not be sufficient to induce TPI activity. Indeed, increasing JA levels by treating *N. miersii* and *N. obtusifolia* with MeJA dramatically increased TPI transcript level and activity [25,26] which is consistent with the hypothesis that the induced TPI activity is dependent on the magnitude and/or duration [30] of JA and JA-Ile induction in *Nicotiana*.

Induced resistance to *M. sexta* and *S. littoralis* is dependent on different HAEs (Figure 5). In *N. attenuata* and *N. pauciflora* induced resistance to *M. sexta* was found after elicitation with C18:3-Glu but not after elicitation with OS_*Sl*_ or OS_*Ms*_. The difference between OS_*Sl*_ and C18:3-Glu induced resistance to *M. sexta* may be due to different elicitors or suppressors within the OS_*Sl*_. However, the difference between resistance induced by OS_*Ms*_ and C18:3-Glu suggests there may be additional components in OS_*Ms*_ which suppress the induced defence in plants, since the C18:3-Glu used for elicitation was at the same concentration as would be found in OS_*Ms*_ (Figure 3A). In both *N. attenuata* and *N. pauciflora*, the induced JA and TPI levels were found to be similar between C18:3-Glu and OS_*Ms*_ treatments (Figures 2 and 4A, 4th and 5th column) suggesting that putative suppressors in *M. sexta* OS may act downstream of JA-signalling or on other signalling pathways that regulate plant metabolism. Indeed a previous study has revealed that in *N. attenuata*, FAC-free OS_*Ms*_ and intact OS_*Ms*_ could induce higher levels of several protein peptides, such as fragments of RuBPCase activase (RCA) compared to FAC alone [31]. In addition RCA recently has been shown to play an important role not only in photosynthesis but also in JA-mediated growth-defence trade-offs [32]. Therefore *M. sexta* may employ some unknown components in its oral secretion to manipulate the growth-defence equilibrium in its host for its own benefit. Cases of insect-suppression of plant-induced defence through components of oral secretion have been reported in a few studies. For example, the OS of *S. littoralis* and *Pieris brassicae* were found to suppress wound-induced defence responses in *Arabidopsis thaliana* [33] and *Leptinotarsa decemlineata* exploits orally-secreted bacteria to suppress defence in tomato [34]. However, studies have also shown that suppression of direct defences by insect oral secretion can also be beneficial for the plant by reducing the cost of direct defences and increasing plant fitness [35,36]. This indicates that in some cases the suppression of induced direct defence by insect oral secretion might be adaptive for both plant and herbivore.

Induced resistance to *S. littoralis* was also shown to be HAE-specific within species (Figure 5). In all four *Nicotiana* species tested, the induced resistance to *S. littoralis* attack was different for each HAE. Furthermore the variation of induced resistance to *S. littoralis* did not appear to correlate with induced JA accumulation. For example in *N. attenuata,* while both OS_*Ms*_ and OS_*Sl*_ treatments showed similar levels of induced JA, the resistance to *S. littoralis* is higher in OS_*Sl*_ induced samples than OS_*Ms*_. The same is true for *N. pauciflora* when treated with C18:3-Glu and OS_*Ms*_, the treatments showed a similar induction of JA accumulation but different induced resistance to *S. littoralis*. These results suggest that the specificity of induced resistance to *S. littoralis* may also be regulated by other signalling pathways or their cross-talk.

In summary *Nicotiana spp.* show highly specific induced-defence responses to different HAEs and this is may be due to differences in their elicitor compositions.

### To which extent does one HAE induce different responses among closely related species?

The same HAE elicited different responses among the six *Nicotiana* species. At the phytohormone level, both C18:3-Glu and OS_*Ms*_ induced JA accumulation in four species. JA accumulation was not induced in *N. miersii* and *N. obtusifolia* which is consistent with previous studies [25,26]. Interestingly in both species, OS_*Sl*_ induced a certain level of JA accumulation (Figure 2). This suggests that these species have intact JA signalling and biosynthesis pathways which can be only specifically activated by some unknown elicitors in OS_*Sl*_. It is therefore reasonable to assume that *N. obtusifolia* and *N. miersii* have lost the ability to perceive C18:3-Glu.

Changes in OS_*Sl*_ perception were also found among *Nicotiana* species. While OS_*Sl*_ induced JA accumulation in the other five species it did not induce JA accumulation in *N. pauciflora* (Figure 2, top row, 5th column). As discussed above, it is likely that *N. pauciflora* has lost its perception of non-FAC, OS_*Sl*_ specific elicitors. Although HAE-induced JA accumulation and JA-mediated defences have been investigated intensively [2,37], the mechanisms of HAE perception remain unknown. The closely related *Nicotiana* species possessing intact JA induction and signalling pathways, but lacking the ability to perceive specific HAEs, offer an ideal system for investigating the molecular mechanisms of HAE perception.

The HAE-induced TPI activity varied among *Nicotiana* species and the variation is correlated with the induced level of JA and JA-Ile (Figure 4B and C). *N. attenuata*, *N. acuminata*, *N. linearis* and *N. pauciflora*, showed high levels of HAE-induced JA accumulation and correspondingly high levels of induced TPI activity (Figure 4A). However *N. obtusifolia* and *N. miersii*, which had little or no induced JA accumulation, showed no induced TPI activity (Figure 4A). This indicates that, at the species level, induced JA and JA-Ile play central roles in the differences in induced TPI activity among different species.

While induced resistance to *S. littoralis* was found in all four *Nicotiana* species tested, only two species showed induced resistance to *M. sexta*. The variation of HAE-induced resistance to *M. sexta* among closely related *Nicotiana* species could be due to differences in their natural histories. For *N. obtusifolia*, although it has low levels of induced defence, it has high levels of constitutive defence, such as TPI and HGL-DTG, which severely affect *M. sexta* performance [38]. Therefore, *N. obtusifolia* may have employed a high constitutive defence strategy rather than an induced defence strategy to defend against herbivores. Perhaps this would be expected of a perennial plant that grows in specialized niches along canyon walls and probably rarely faces the type of strong intra-specific competition which is thought to have selected for the elaborate induced defence system of *N. attenuata* [35].

Interestingly, *N. miersii* has both low constitutive and induced defence and is the fastest growing plant among the six species studied (glasshouse observation). This is consistent with *N. miersii*’s low level of TPI activity (Figure 4A) [25], as high TPI activity has been shown to slow plant development and reduce fitness in *N. attenuata* [39]. In addition the level of constitutive and induced defence compounds, such as nicotine, nornicotine, caffeoylputrescine and HGL-DTG were very low in *N. miersii* [25]. This is consistent with the hypothesis that this species may have evolved a high growth - low defence strategy. Indeed under low herbivore pressure, selection on plants will favour a high competitive ability, such as that provided by high growth rate [40,41]. Although little is known about the herbivore composition of its natural habitat, we predict that *N. miersii* has lost its induced defence against *M. sexta* due to low herbivore pressure, or an exceptionally high level of intra-specific competition. Thus, the variation of induced defences among *Nicotiana* species is thought to result from differences in their natural histories and selection pressures. Taken together, our data show that one HAE can induce different defence responses among closely related species at all different levels. This indicates that induced defences can evolve rapidly in *Nicotiana*. Indeed, even within the species of *N. attenuata,* two genotypes showed different OS_*Ms*_ induced early defence signalling [42]. The analysis of large numbers of *N. attenuata* isolates collected from different natural populations also suggested that OS_*Ms*_ induced JA and JA-Ile levels were indeed variable but largely followed a Gaussian distribution within *N. attenuata* (Additional file 2 and Li. et al. Submitted). However, the inter-species variations that were found in this study are much greater than intra-species variation, at least at the level of OS_*Ms*_ induced JA and JA-Ile levels (Additional file 2).

### Does one induced defence affect insect herbivores differently?

**Figure 6:**
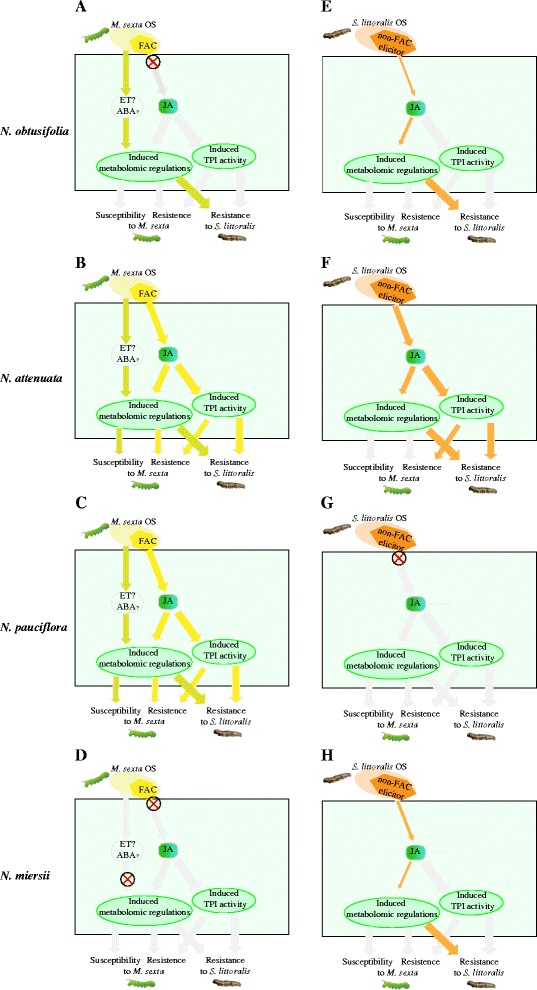
**A model summarizing the evidence for specificity of HAE-induced defences in four** ***Nicotiana*** **species.** The coloured arrows indicate the interaction between two components. The colours refers to different HAEs and the filling amount of arrow indicates the strength of an interaction. Grey arrow indicates that no interaction was found. Each panel represents the induced defence model in different species. Red cross in the circle indicates a putative loss of function mutation. **A**-**D** and **E**-**H** refer to the *M. sexta* OS **(A-D)** and *S. littoralis* **(E-H)** induced defence responses in *N. obtusifolia* **(A and E)**, *N. attenuata* **(B and F)**, *N. pauciflora* **(C and G)** and *N. miersii* **(D and H)** respectively. JA: jasmonic acid; ET: ethylene; ABA: abscisic acid; OS: oral secretion; TPI: trypsin proteinase inhibitor.

In *Nicotiana spp.*, while C18:3-Glu induced defences negatively affected the growth of both *M. sexta* and *S. littoralis*; the other two HAEs could only reduce the growth rate of *S. littoralis* (Figures 5 and 6). This may be due to differences in the susceptibility of the specialist *M. sexta* and generalist *S. littoralis* to induced plant defences. Because of the long co-evolutionary history between specialists and their hosts, specialists are more able to tolerate and detoxify their host’s defence compounds than generalists [20]. Therefore it is likely that while many different induced defence responses can affect the growth rate of *S. littoralis*, the growth rate of *M. sexta* can only be affected by specific, or combinations of specific, defence traits. This hypothesis is supported by the correlation between the induction of TPI (a specialised *Nicotiana* defence trait) and the increased efficacy of defence against *M. sexta* and *S. littoralis*. The multiple-domain TPI gene has been shown to be specific to the *Nicotiana* genus and it is reasonable to posit that it evolved as a consequence of a plant-insect arms race [43,44]. The induced resistance to *M. sexta* shown by C18:3-Glu treated *N. attenuata* and *N. pauciflora* was correlated with induced TPI activity (Figures 4A and 5) indicating that induction of TPI might be necessary (but not sufficient) to reduce the growth of *M. sexta*. However species such as *N. obtusifolia* showed induced resistance to *S. littoralis* but did not show TPI activity after elicitation with OS_*Ms*_ and OS_*Sl*_ (Figures 5 and 6). This suggests that TPI induction is not required to reduce the growth rate of *S. littoralis*. In summary our data suggests that same induced defence response affects specialist and generalist insect herbivores differently.

## Conclusions

Our systematic investigation of HAE-induced defence in *Nicotiana* showed that in these species induced defence is highly specific at all three analysed perspectives: a single *Nicotiana* species can show different defence responses to different HAEs; one HAE can induce different responses among closely related species and the effect of one HAE-induced defence response differs among herbivorous insects. Furthermore, the analysis also indicates that HAE-induced defence can evolve rapidly, because the same HAE can induce divergent responses among closely related *Nicotiana* species*.* These closely related species showing distinct HAE-induced defences are therefore an ideal system for the future study of the molecular mechanisms and evolutionary divergence of herbivore-induced defence.

## Methods

### Plant growth and sample treatments

The seeds of different *Nicotiana* species were either originally collected from natural populations or obtained from the US *Nicotiana* Germplasm Collection, North Carolina. *Nicotiana attenuata* Torr. Ex S. Watson and *N. obtusifolia* seeds were originally collected in Utah (USA) and inbred in the glasshouse for 30 generations and 1 generation respectively. The other four *Nicotiana* species were originally obtained from the US *Nicotiana* Germplasm Collection and inbred for one generation in the glasshouse. The detailed accession numbers and availability of seeds for all plant species used in our experiments are provided in the Additional file 3. All seeds were germinated according to the *N. attenuata* germination protocol [45]. Ten-day-old seedlings were planted into soil in Teku pots (Waalwijk) and, once established, transferred to 1 L pots in soil and grown in a York Chamber under a 16/8 h light/dark, 26°C, and 65% relative humidity regime until they were in the rosette stage.

*M. sexta* and *S. littoralis* oral secretions (OS) were collected on ice from larvae reared on *N. attenuata* plants until third to fifth instar as previously described [21]. To analyse the FAC components in the OS each sample was diluted 1:100 (OS_*Ms*_) or 1:10 (OS_*Sl*_) with 15% methanol and analysed directly on a high-performance liquid chromatograph-mass spectrometer (HPLC-MS) (1200 L LC-MS, Varian, Palo Alto, CA, USA) [21]. FAC-free OS_*Sl*_ was obtained using the method described in [21]. The synthetic C18:3-Glu used in this study was diluted to 138 ng μL^-1^, which is the same concentration as that found in OS_*Ms*_.

To simulate herbivore attack one leaf of each plant was wounded with a pattern wheel and 20 μL of 1:5 diluted OS_*Ms*_ or OS_*Sl*_ or C18:3-Glu or water was added to the puncture wounds. Leaf samples were collected at 0 h, 0.5 h, 1 h and 2 h for phytohormone analysis. Samples for trypsin proteinase inhibitor (TPI) assays were collected 24 h after treatment. The middle vein of the leaf was excised. All samples were collected in 2 mL Eppendorf tubes, flash frozen in liquid nitrogen then stored at -80°C until analysed.

### Phytohormone and TPI activity measurements

Phytohormones were quantified as previously described [15]. In brief, the ground tissues were extracted with ethyl acetate spiked with 100 ng/mL of 9,10-dideutero-9,10-dihydro jasmonic acid (JA-D2), 20 ng/mL jasmonoyl isoleucine (JA-Ile-13C6), 20 ng/mL hexadeutero abscisic acid (ABA-D6) and 20 ng/mL 3,4,5,6-tetradeutero salicylic acid (SA-D4) as internal standards (ISs). Then the extracts were re-suspended in 70% methanol and the phytohormone content was analysed via HPLC-MS (1200 L LC-MS, Varian, Palo Alto, CA, USA) [15]. TPI activity was analysed in 100 mg of leaf tissue with a radial diffusion assay as described by Van Dam et al [46].

### Induced resistance detached leaf assay

To prevent insect feeding effects that may mask HAE-induced responses, the HAE-induced resistance to both *M. sexta* and *S. littoralis* was measured through bioassays using detached leaf feeding. *M. sexta* were obtained from in-house colonies and *S. littoralis* were obtained from Syngenta (Stein, Switzerland). All bioassays were carried out in an insect chamber (Snijders Scientific, Tilburg, The Netherlands) at 16/8 h light/dark, 26°C, 80% humidity and 100 μmol m^-2^ s^-1^ light intensity. The bioassays for the four treatments in each species were performed at the same time of day in the same insect chamber. For each plant species, rosette stage leaves (leaves at node +1) were wounded and treated with OS_*Ms*_, OS_*Sl*_, C18:3-Glu or water (control) at 11 am. After 24 h, the treated leaves were cut and mounted in 2 mL Eppendorf tubes filled with moist cotton, then placed in a vertical position inside clear polystyrene food boxes. The leaves were replaced every two days with new leaves from plants that were treated with the same HAE 24 h previously. To ensure all insect larvae were at a similar developmental stage and with the same body mass at the start of each bioassay, the newly hatched neonate (*M. sexta*) or first instar larvae (*S. littoralis*, fed on artificial diet) were first placed on the untreated leaves of the test species for 48 h. Then the neonates were weighed and 20 neonates with similar mass (6–10 mg and 1.8–3.3 mg for *M. sexta* and *S. littoralis* respectively) were selected and used for the bioassays. For *M. sexta* the bioassays were stopped after 5 days. For *S. littoralis*, the bioassays lasted 6 days, except the assays on *N. pauciflora* and *N. miersii* which lasted only 5 days because the leaves of these species are not large enough to feed *S. littorals* beyond this time. At the end of the bioassay all surviving larvae were weighed. To make the induced resistance comparable among different species the body mass was normalized to the average larval body mass of the control group (fed on Wounding + Water treated leaves) for each bioassay.

### Statistical analysis

All statistical analyses and non-targeted metabolomic analyses were performed in R version 3.0.1 [47]. The significance of phytohormone and TPI activity induced by different HAEs was assessed by parametric or non-parametric tests after normality testing of the data distribution by the Shapiro test. For parametric tests ANOVA and post hoc Tukey honest significant difference (HSD) tests were carried out by using the “aov” and “TukeyHSD” functions. For non-parametric tests, Kruskal-Wallis and associated multiple-comparison tests were carried out by using the “kruskalmc” function in the “pgirmess” library. The significance of induced resistance between HAE and control was assessed by the Student’s-t test or the Mann–Whitney U test after normality testing of data distribution by the Shapiro test.

## Availability of supporting data

The data sets supporting the results of this article are available in the Labarchives repository: http://doi.org/10.6070/H4DF6P6W.

## Additional files

Additional file 1:
**C18:3-Glu supplemented** ***S. littoralis*** **OS (OS**_***Sl***_ **) induced JA accumulations in** ***N. pauciflora*** **to levels equivalent to those induced by** ***M. sexta*** **OS (OS**_***Ms***_ **).** JA was measured at two hours after the wounding plus OS treatment when *N. pauciflora* showed the highest induced JA accumulation. OS_*Sl*_ was supplemented with an amount of C18:3-Glu equivalent to that found in *M. sexta* OS (OS_*Ms*_). NS indicates no statistical difference was found in comparison to control (wounding + water). Letter indicates statistical significance was found in comparison to control (*p* < 0.05, *post hoc* Tukey honest significant test after ANOVA). Additional file 2:
**The variation of OS**_***Ms***_ **induced JA accumulations among closely related** ***Nicotiana*** **species is much greater than different** ***N. attenuata*** **accessions.** The level of JA and JA-Ile accumulation was measured at one hour after OS_*Ms*_ treatment. In order to compare the intra-species and inter-species variation, we normalized the JA level to *N. attenuata* UT accession (30th in bred), which was used in both datasets. Red color refers to closely related *Nicotiana* species that were used in this study, blue color refers to different accessions of *N. attenuata* collected from natural population. The detailed sample information of different *N. attenuata* is included in another publication (Li. et. al. submitted). A: the density plot of OS_*Ms*_ induced JA level; B: the distribution of OS_*Ms*_ induced JA-Ile level. Additional file 3:
**The stock centre numbers and availability of plants that were used in this study.**

## Abbreviations

HAE: Herbivore associated elicitor
OS: Oral secretion
FAC: Fatty acid amino acid conjugate
JA: Jasmonic acid
TPI: Trypsin protease inhibitor
