# Supplementary figures and images for: Herbivore associated elicitor-induced defences are highly specific among closely related Nicotiana species
Source: BMC Plant Biol. 2015 Jan 16;15:2. doi: 10.1186/s12870-014-0406-0 (PMC4304619; doi:10.1186/s12870-014-0406-0)

**JA (ng/g FW leaf)**

2000  
1000  
0

**Water**

**OS<sub>Sl</sub>**

**C18:3-Glu + OS<sub>Sl</sub>**

**C18:3-Glu**

**OS<sub>Ms</sub>**

**Wounding +**

**A**

**A**

**B**

**B**

**B**

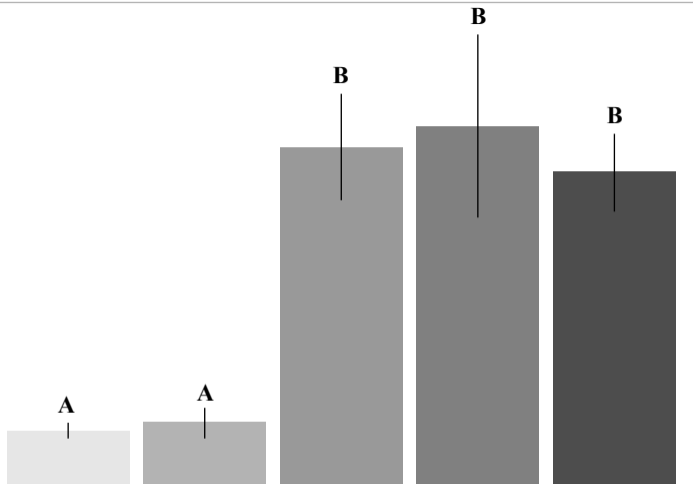

Supplement: Additional file 1: — C18:3-Glu supplemented S. littoralis OS (OS Sl ) induced JA accumulations in N. pauciflora to levels equivalent to those induced by M. sexta OS (OS Ms ). JA was measured at two hours after the wounding plus OS treatment when N. pauciflora showed the highest induced JA accumulation. OSSl was supplemented with an amount of C18:3-Glu equivalent to that found in M. sexta OS (OSMs). NS indicates no statistical difference was found in comparison to control (wounding + water). Letter indicates statistical significance was found in comparison to control (p < 0.05, post hoc Tukey honest significant test after ANOVA). [file 12870_2014_406_MOESM1_ESM.pdf]

A

*M. sexta* OS induced JA level

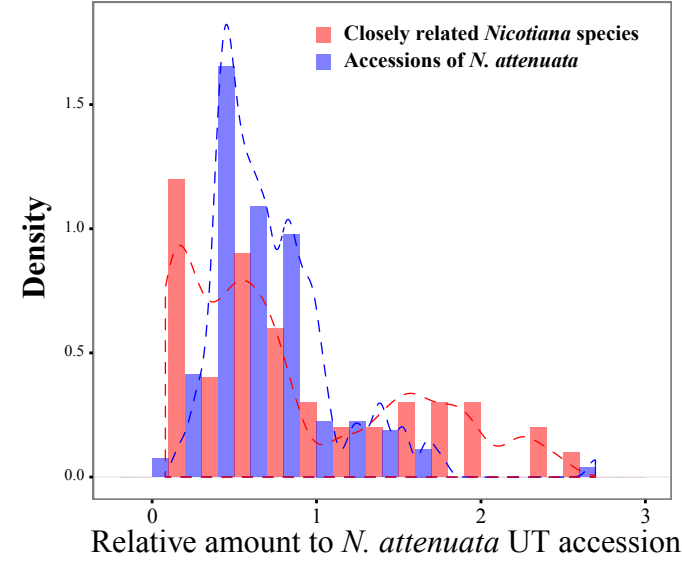

B

*M. sexta* OS induced JA-Ile level

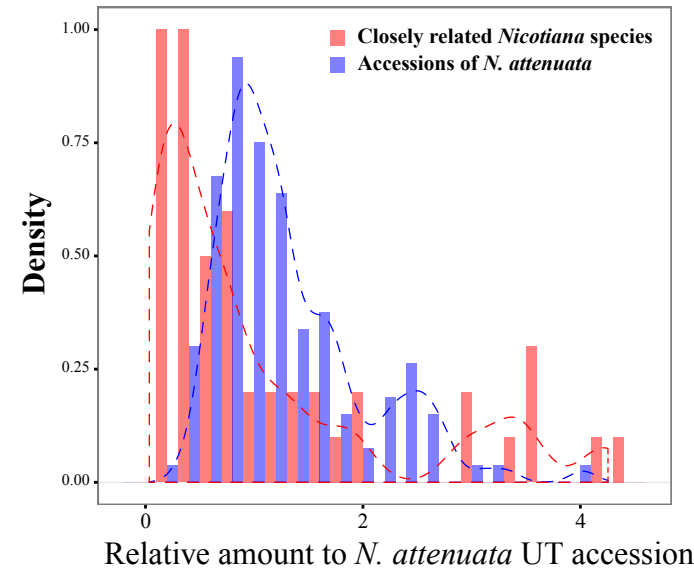

Supplement: Additional file 2: — The variation of OS Ms induced JA accumulations among closely related Nicotiana species is much greater than different N. attenuata accessions. The level of JA and JA-Ile accumulation was measured at one hour after OSMs treatment. In order to compare the intra-species and inter-species variation, we normalized the JA level to N. attenuata UT accession (30th in bred), which was used in both datasets. Red color refers to closely related Nicotiana species that were used in this study, blue color refers to different accessions of N. attenuata collected from natural population. The detailed sample information of different N. attenuata is included in another publication (Li. et. al. submitted). A: the density plot of OSMs induced JA level; B: the distribution of OSMs induced JA-Ile level. [file 12870_2014_406_MOESM2_ESM.pdf]
